# Acute Myocardial Infarction in Young Individuals: Demographic and Risk Factor Profile, Clinical Features, Angiographic Findings and In-Hospital Outcome

**DOI:** 10.7759/cureus.45803

**Published:** 2023-09-22

**Authors:** Iva N Dimitrova

**Affiliations:** 1 Cardiology, University Hospital “Prof. Alexandar Tschirkov”, Medical University of Sofia, Sofia, BGR

**Keywords:** myocardial infarction, outcome, coronary artery angiography, cardiovascular risk factor, young

## Abstract

Background: Cardiovascular diseases (CVDs) are the leading cause of global mortality and disability. Acute myocardial infarction (AMI) in young individuals is a rare condition but can cause devastating socioeconomic and psychological consequences for both the patient and their family and an economic burden for the government. There is a paucity of data concerning the specific profile of these young patients in Bulgaria, a country with a high burden of cardiovascular morbidity and mortality. Therefore, the aim of the present study was to assess the baseline characteristics, demographic and risk factor profile, clinical features, angiographic findings and in-hospital outcomes of young patients with AMI. Additionally, these data were compared to those of the older age group.

Methods: Retrospective data on 172 patients treated for AMI in “Prof. Alexandar Tschirkov”, Bulgaria, from January 2018 to December 2021 were collected for the purpose of this study. Baseline characteristics, risk factor profile and clinical and angiographic features were compared in young (≤45 years) and older patients (>45 years).

Results: Males were significantly predominant in the two age groups (p < 0.01), with an obviously increasing proportion of females in the older age group. Young patients were more likely to be smokers (55.7% vs. 28.8%; p=0.001); in contrast, hypertension (70.5% vs. 91.9%; p <0.001) and obesity (16.4% vs. 31.5%; p=0.031) were more prevalent in older patients. Anterior localization of myocardial infarction (MI) was most common in the two age groups (47.5% vs. 41.4%), respectively. Regarding the time delay from symptom onset to first medical contact, we found that young patients tended to present earlier than their older counterparts. Young patients had a higher incidence of single vessel disease (SVD) (49.2%) and nonobstructive coronary disease (NOCD) (11.5%) than older patients. Young patients with AMI had a lower in-hospital morbidity rate than older patients, but the in-hospital mortality, although lower, was not significantly different. A high prevalence of modifiable cardiovascular risk factors (RFs), such as smoking, dyslipidemia and arterial hypertension (AH), among the young group, less evolved CAD and similar high procedural success between age groups were established. Approximately 30% of young patients tend to present late in the hospital. The in-hospital mortality in the young population was lower than that in the older population but was still relatively higher than that previously reported.

Conclusion: The reported high prevalence of modifiable RFs and late presentation of young patients with AMI highlight the need for early recognition of these RFs, better prevention, deployment of educational programs, easy access to health care and high awareness of clinicians to reduce disability and mortality from CVD.

## Introduction

Cardiovascular diseases (CVDs) are the leading cause of global mortality and disability. According to the Global Burden of Cardiovascular Diseases and Risk Factors, 1990-2019, there is a trend of a constantly increasing number of CVD deaths, from 12.1 million in 1990 to 18.6 million in 2019 [1]. In particular, ischemic heart disease (IHD) causes 49.2% of all CVD deaths [1]. Acute myocardial infarction (AMI) is uncommon in young individuals, and its incidence varies between 2% and 10% [2-4]. Despite the better prognosis at younger ages and its relatively low incidence, AMI at young ages can be a life-threatening condition and cause long-term disability. Moreover, it can cause socioeconomic and devastating psychological consequences for both the patient and their family. Therefore, to modify some risk factors (RFs), clinical interest in AMI in young patients is increasing.

Several studies have shown that young patients with AMI have different clinical presentations, RFs, angiographic profiles and prognoses compared with their older counterparts [5,6]. Rubin and Borden [5] indicated that young patients with CVDs are predominantly smokers and obese males with a positive family history. Moreover, in these patients, nontraditional RFs and etiologies, such as abnormal coronary arteries, connective soft tissue and autoimmune diseases and illicit drug use, are more frequent causes of AMI than in older patients. Chen et al. [6] confirmed the essential role of these RFs but also noted that dyslipidemia and ST-elevation AMI are commonly observed in patients younger than 45 years. Young AMI patients were more likely to have single-vessel involvement and a lower degree of left ventricular dysfunction. Finally, both studies concluded that despite benign prognoses, the modification of RFs in young patients is crucial [5,6].

Eastern Europe is a region with one of the highest age-standardized rates for disability-adjusted life years (DALYs) [1]. However, there is scarce information concerning the specific profile of the abovementioned characteristics in young patients with AMI in this region. Therefore, the aim of this study was to describe demographic and baseline characteristics, RFs, angiographic features, in-hospital complications and outcomes in young AMI patients (≤45 years). Additionally, the differences between these patients and older patients (>45 years) were analyzed.

## Materials and methods

This study was conducted in “Prof. Alexandar Tschirkov”, Bulgaria, from January 2018 to December 2021. Retrospective data on 172 patients treated for ST-elevation myocardial infarction (STEMI) and non-ST-elevation myocardial infarction (NSTEMI) according to the criteria of the Fourth Universal Definition of Myocardial Infarction (2018) were included in this study [7]. Participants were divided into two groups: group 1 (younger group): all consecutive patients (n=61) between 18 and 45 years in this period admitted for AMI; and group 2 (older group) (n= 111): patients >45 years with AMI selected from the hospital electronic database in the abovementioned period using the platform Research Randomizer in an absolutely random way to achieve statistical authenticity.

There is no universal definition of “young” regarding the diagnosis of AMI, but most studies use the age cutoff of 40-45 years (ranging between 30 and 55 years) [2-5]. Therefore, we accept our “young” patients to be under 45 years old. For the present analysis, only patients with type 1 and type 2 AMI were included. In brief, type 1 AMI is caused by atherothrombotic coronary artery disease, complicated by plaque rupture or erosion; type 2 is caused by an acute imbalance between oxygen supply and demand to the heart muscle; type 3 is a fatal AMI with no rise or fall in cardiac biomarkers levels; type 4 occurs during or shortly after coronary angioplasty or stenting; and type 5 AMI occurs during or shortly after cardiac surgery [7].

All patients underwent coronary angiography with or without percutaneous coronary intervention (PCI) after signing informed consent. Exclusion criteria were as follows: 1) patients <18 years old; 2) patients with AMI type 3-5 according to the definition; 3) patients who did not undergo coronary angiography; and 4) missing or incomplete information concerning the aims of the study. Data about baseline, clinical characteristics, RFs, echocardiography and angiographic findings, length of hospital stay, complications and in-hospital outcome were retrospectively collected and analyzed from the hospital electronic database, including echocardiography protocols, angiography reports, discharge documents and outpatient clinic reports.

Demographic characteristics included age and gender. The traditional RFs for each patient were evaluated. Diabetes mellitus (DM) was defined as fasting plasma glucose ≥7.0 mmol/L, hemoglobin A1c (HbA1c) ≥6.5% or outpatient diagnosis of DM. Arterial hypertension (AH) was defined as a systolic blood pressure >140 mmHg and/or a diastolic blood pressure >90 mmHg and/or the use of antihypertensive drugs. Dyslipidemia was defined as a total plasma cholesterol >5.2 mmol/l; low-density lipoprotein (LDL) ≥2.6 mmol/l; triglycerides ≥1.7 mmol/l; high-density lipoprotein (HDL) ≤1 mmol/l for men and ≤1.3 mmol/l for women or the use of cholesterol-lowering drugs. A family history of coronary heart disease was defined as any first-degree relative younger than 55 years who was affected by IHD. Patients who currently smoke or who stopped less than a year ago are defined as smokers, and the rest are defined as nonsmokers. Obesity was defined by BMI ≥30 kg/m^2^. Information about illicit drug abuse was obtained. The personal history of IHD, such as previous myocardial infarction (MI) or coronary revascularization, was also investigated.

Left ventricular ejection fraction (LVEF) was assessed by transthoracic echocardiography using the Simpson method. We defined patients with preserved LVEF ≥50%, mildly reduced LVEF 41%-49% and reduced LVEF ≤40%. All patients underwent coronary angiography with subsequent PCI if necessary via a transradial (preferred) or transfemoral approach. The angiographic and PCI data were retrieved from the procedural report and were reviewed by an interventional cardiologist expert. Obstructive coronary disease was defined as at least a 70% narrowing in the diameter of the three major coronary arteries or their branches or ≥50% reduction in the left main artery (LMA). According to the number of vessels involved, coronary artery disease was categorized as single vessel disease (SVD), double vessel disease (DVD) or multivessel disease (MVD). Patients with normal coronary arteries or minimal atherosclerotic involvement are qualified as having nonobstructive coronary disease (NOCD). Infarct-related artery (IRA) and LMA involvement were noted. The number of deployed stents was determined, and the final result after PCI was evaluated using Thrombolysis in Myocardial Infarction (TIMI) grade flow.

In-hospital complications, such as rhythm and conductive disturbances requiring medical or instrumental therapy (ventricular tachycardia or ventricular fibrillation/high-grade or complete atrioventricular block with or without a temporary pacemaker), acute heart failure and Killip class, cardiac arrest, mechanical complications of AMI, postinfarction angina during the hospital stay, reinfarction and death are reported. The length of hospital stay was also analyzed. Concerning statistical analysis, all data were checked and analyzed using Statistical Package for the Social Sciences (SPSS) software for Windows version 20.0. Continuous variables are expressed as the mean and standard deviation. Comparison of categorical variables between groups was performed using the chi-square or Fisher’s exact test, and a p-value ≤0.05 was considered statistically significant.

## Results

Demographic characteristics and risk profile

The youngest patient in the study population was 23 years old, and the oldest was 89 years old. The average age of patients in the young group was 39.74 (±4.97) years compared with 67.21 (±10.18) years in the older age group. Males were significantly predominant in the two age groups: group 1: 56 males and five females (91.8% vs. 8.2%; p < 0.01) and group 2: 78 males and 33 females (70.3% vs. 29.7%; p<0.01), with an obviously increasing proportion of females in the older age group.

The RF profile was different between the two groups. The most prevalent RFs in the overall sample were dyslipidemia (88.4%), followed by AH (84.3%) and smoking (38.4%). There was a statistically significant difference between the prevalence of three RFs between the two age groups. Young patients were more likely to be smokers (55.7% vs. 28.8%; p=0.001), and patients in group 2 were more likely to have AH (70.5% vs. 91.9%; p<0.001) and to be obese (16.4% vs. 31.5%; p=0.031). A higher number of the patients in group 2 had DM, but the difference was not statistically significant (18.0% vs. 29.7%; p=0.093). Two patients from group 1 reported illicit drug abuse and none of the older patients. Only 3.3% from the younger group and 0.9% from the older group had none of the abovementioned traditional cardiovascular RFs. Patients aged ≤45 years were less likely than older patients to have a history of previous MI and previous revascularization, with statistical significance for revascularization (p<0.001) (Table 1).

**Table 1:** Demographic characteristics and risk profile AH, arterial hypertension; DM, diabetes mellitus; CAD, coronary artery disease; MI, myocardial infarction. Values presented as number (n); % and mean±SD.

Demographic characteristics and risk profile	≤45 years (n=61)	>45 years (n=111)	p-value
Age (years)	39.74 (±4.97)	67.21 (±10.18)	
Male gender	56 (91.8%)	78 (70.3%)	0.001
AH	43 (70.5%)	102 (91.9%)	<0.001
Dyslipidemia	54 (88.5%)	98 (88.3%)	0.963
DM	11 (18.0%)	33 (29.7%)	0.093
Smoking	34 (55.7%)	32 (28.8%)	0.001
Family history of CAD	17 (27.9%)	29 (26.1%)	0.805
Obesity	10 (16.4%)	35 (31.5%)	0.031
Substance use	2 (3.3%)	0 (0.0%)	0.124
Previous MI	4 (6.7%)	15 (13.5%)	0.174
Previous revascularization	2 (3.3%)	27 (24.3%)	<0.001

Presentation and clinical features

The majority of patients in group 1 and group 2 had symptoms of typical chest pain, 90.16% vs. 85.6%, respectively (p=0.534). Other symptoms were nausea, sweating, vomiting, shortness of breath, syncope, weakness and palpitation. Anterior AMI was the most common in the two age groups (47.5% vs. 41.4%), respectively.

Regarding the time delay from symptom onset to first medical contact, we found that young patients tended to present earlier than their older counterparts. Forty-three percent of the patients in group 2 and 27.9% of the patients in group 1 had more than six hours of symptom delay (p=0.047). In a subgroup analysis, we did not observe significantly longer delays in women than in men in the two age groups (both p=0.6). As expected, more patients in the older age group presented with Killip class >II, and we observed borderline significance (p = 0.057).

Reduced LVEF ≤40% was more common in patients in group 2 (19.7% vs. 21.6%; p= 0.932), but there was no significant difference between groups. Оlder patients tended to have a higher prevalence of mitral insufficiency ≥II grade (p<0.001). The mean peak values of creatine phosphokinase (CPK), CPK-MB and high-sensitivity troponin I (hsTN-I) during hospitalization were in similar ranges in both groups (Table 2).

**Table 2:** Presentation and clinical features AMI: acute myocardial infarction, LVEF, left ventricular ejection fraction; CPK, creatine phosphokinase; hsTN-I, high-sensitivity troponin I.

Presentation and clinical features	≤45 years (n=61)	>45 years (n=111)	p-value
Symptom presentation			
Chest pain	55 (90.2%)	95 (85.6%)	0.534
No chest pain	18 (29.5%)	16 (14.4%)	
AMI localization			
Anterior	29 (47.5%)	46 (41.4%)	0.822
Inferior	17 (27.9%)	38 (34.2%)	
Lateral	6 (9.8%)	12 (10.8%)	
Inferolateral	9 (14.8%)	15 (13.5%)	
Time delay			
<6 h	44 (72.1%)	63 (56.8%)	0.047
>6 h	17 (27.9%)	48 (43.2%)	
Killip class > II	3 (4.9%)	16 (14.4%)	0.057
LVEF (assessed by echocardiogram), %			
Preserved LVEF ≥50%	38 (62.3%)	66 (59.5%)	0.932
Mildly reduced LVEF 41%-49%	11 (18.0%)	21 (18.9%)	
Reduced LVEF ≤40%	12 (19.7%)	24 (21.6%)	
Mitral insufficiency ≥ II grade	1 (1.6%)	30 (27.0%)	<0.001
Mean peak values of cardiac biomarkers			
CK, U/l	2657.08	997.66	0.995
CK-MB, U/l	161.37	100.52	0.987
hsTN- I, ng/ml	17.96	16.96	0.546

Procedural characteristics

A total of 172 patients with AMI underwent coronary angiography and a subsequent PCI when appropriated according to the current recommendations. The radial approach was preferred in the two age groups (95.1% vs. 85.6%; p= 0.057). Analysis of coronary angiogram showed that the left anterior descending artery (LAD) was the most commonly involved IRA in group 1 (40.9%), followed by the right coronary artery (RCA) in 27.9% of cases; in contrast, in group 2, the rate was 37.8% for RCA and 36.9% for LAD. LMA involvement was significantly higher in patients ≥45 years (1.6% vs. 14.4%; p=0.007).

Single-vessel involvement was the most common angiographic pattern in our study among patients in group 1 (49.2%), followed by DVD (27.9%) and MVD (11.5%). For group 2, the most prevalent was MVD (46.8%). For SVD and MVD, the established differences were significant between groups. NOCD was seen in 11.5% of young patients and 1.8% of older patients (p=0.006). The mean number of stents deployed was significantly higher in group 2. Procedural success, defined by the final TIMI III flow in the IRA, was over 90% in both groups (Table 3).

**Table 3:** Procedural characteristics IRA, infarct-related artery; LAD, left anterior descending artery; RCA, right coronary artery; Cx, circumflex artery; LMA, left main artery; SVG, saphenous vein graft; RIM, ramus intermedius artery; SVD, single vessel disease, DVD, double vessel disease; MVD, multivessel disease; NOCD, nonobstructive coronary disease; PCI, percutaneous coronary intervention; TIMI: Thrombolysis in Myocardial Infarction; LIMA: left internal mammary artery.

Procedural characteristics	≤45 years (n=61)	>45 years (n=111)	p-value
Radial approach	58 (95.1%)	95 (85.6%)	0.057
IRA			
LAD	25 (40.9%)	41 (36.9%)	0.601
RCA	17 (27.9%)	42 (37.8%)	0.18
Cx	11 (18.03%)	19 (17.11%)	0.87
LMA	1 (1.6%)	4 (3.6%)	0.46
LIMA	0 (0.0%)	1 (0.9%)	
SVG RCA	0 (0.0%)	1 (0.9%)	
SVG RIM	0 (0.0%)	1 (0.9%)	
Number of diseased vessels			
SVD	30 (49.2%)	25 (22.5%)	<0.001
DVD	17 (27.9%)	32 (28.8%)	0.894
MVD	7 (11.5%)	52 (46.8%)	<0.001
NOCD	7 (11.5%)	2 (1.8%)	0.006
LMA involvement	1 (1.6%)	16 (14.4%)	0.007
Post-PCI TIMI III flow grade	53 (91.4%)	102 (93.6%)	0.754
Mean number of stents deployed	1.0	1.23	0.045

In-hospital morbidity and mortality

**Figure 1:**
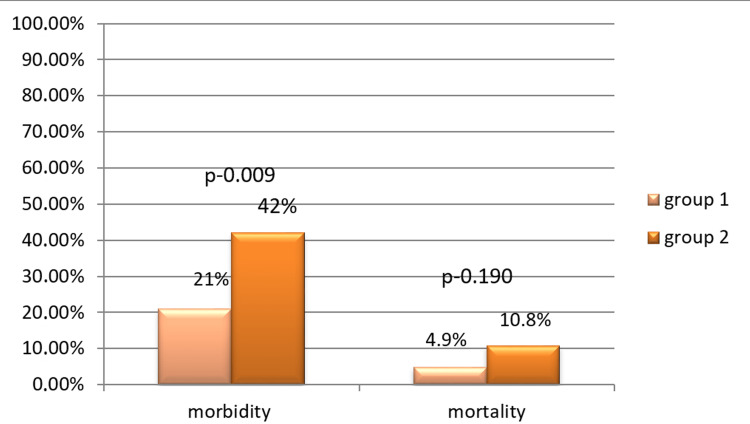
In-hospital morbidity and mortality

The overall in-hospital outcomes are represented in Figure 1.

The mean length of hospital stay was similar between groups: 4.16 days for group 1 and 4.48 days for group 2 (p=0.204). Approximately 13 (21%) young patients and 47 (42%) older patients developed complications during the AMI phase (p=0.009). The most common complications in the young age group were ventricular tachycardia or ventricular fibrillation (4.9%), cardiogenic shock (4.9%), resuscitated cardiac arrest (4.9%) and postinfarction angina (4.9%). Cardiogenic shock (13.5%), followed by resuscitated cardiac arrest (11.7%) and high-grade or complete atrioventricular block or temporary pacemaker implantation (9.0%) were most common in the older group. No cases of reinfarction were observed during the hospital stay in either group. Young patients had lower in-hospital mortality (4.9% vs. 10.8%, p=0.190) than older patients, but the difference was not statistically significant. In the subgroup analysis, we did not find a significant difference concerning in-hospital mortality between males and females.

## Discussion

This retrospective analysis focused on the between-group comparisons of demographic, baseline, clinical characteristics, risk profile, angiographic features and in-hospital outcomes of AMI according to age. We aimed to reveal a specific profile of young patients with AMI who underwent invasive strategy treatment. Despite the devastating consequences that CVD may have, especially in young individuals and the high incidence of cardiovascular (CV) morbidity and mortality in Eastern Europe, there is a lack of data concerning this topic in our region.

A total predominance of male sex in the current study in the young patient group (91.8%) was observed, with the proportion decreasing with increasing age, confirming the existing literature data [8]. This may be due to the protective effects of estrogens in preventing atherosclerosis in young women. The male prevalence has been suggested as a potential reason for delaying treatment in young women presenting with AMI [9].

Among the RFs for CAD, the most prevalent in young AMI individuals were dyslipidemia, AH and smoking, followed by a family history of CAD. Young patients were more likely to be current smokers at the time of study enrollment (p=0.001). AH was more frequent in older patients (p<0.001), as was obesity (p=0.031). We observed borderline significance for the prevalence of DM in group 2 (p=0.093).

The proportion of dyslipidemia in both groups was higher than that reported in other studies, without reaching statistical significance between groups [10-12]. Possible reasons for this discrepancy may be the diversity of “dyslipidemia” definitions, genetic factors, unhealthy behavior habits and underestimation of primary prophylaxis in our country. Despite controversial data concerning the role of dyslipidemia as RF, especially in young patients, Lei and Bin [8] in their meta-analysis defined it as a major RF and especially high serum levels of LDL cholesterol. They found that young AMI patients had higher levels of serum triglycerides (TG), LDL and total cholesterol (TC) and lower serum HDL levels than older AMI patients. These facts support the need for early identification of dyslipidemia at a younger age, correction of the lipid profile and special emphasis on serum HDL cholesterol levels [8]. However, those results, indicating a high level of this RF in the study population, are similar to those by Chan et al. [13] in a single-center, registry-based study in Singapore.

AH was also a very common RF in our study cohort, reaching high levels in 70.5% of patients in group 1 and in 91.9% of patients in group 2 (p<0.001). Although it was significantly prevalent in older patients, its extent among the young group deserves attention. According to most authors, DM and AH are priorities for the older age group [2,4,6,8]. In the VALIANT study, according to Anderson et al. [14], although AH predominated in older patients with AMI, the adjusted relative risk for this RF was higher in young patients with regard to clinical outcomes. Therefore, early diagnosis of AH and its treatment can reduce the incidence of AMI in young people [8].

Smoking seemed to be the major traditional RF for young AMI patients according to most authors [2,8,10,11]. Yandrapalli et al. [10], in a retrospective cohort analysis of young AMI individuals in the United States, reported rates of tobacco use in 56.8% of patients, which was similar to our results (55.7%). The majority of studies presented an even higher rate of smoking in young persons with acute coronary syndrome, ranging from 62% to 90% [2,13,15-17].

Smoking, as a traditional RF, may have multiple mechanisms in the development of CAD: it plays a role not only in atherosclerosis, contributing to early coronary incidents, but also in thrombogenesis and a higher tendency for coronary vasospasm. Smoking contributes to endothelial dysfunction, which increases platelet aggregation and blood viscosity. These mechanisms may partly explain the high frequency of NOCD found in young AMI smokers [18].

A significant association was observed between age and smoking and age and AH with respect to outcome, with the adjusted relative risk due to these factors being most pronounced in young patients. Of these RFs, only smoking was prevalent in young patients and was associated with a greater risk of post-AMI events in these patients compared to the older age group. The risk attributable to smoking decreased with age [14].

The high prevalence rate of smoking, as a modifiable risk factor in our young group of patients, highlights the need for effective and aggressive primary prevention and government programs aiming to increase the awareness of young individuals to reduce the burden of CVD. Only two patients in group 1 reported illicit drug abuse. However, in the current study, no test for narcotic substances was used, so we supposed that the number may be higher.

Typical chest pain was the most common symptom in the study cohort but less frequently with increasing age. Consistent with previous studies, young patients had a significantly shorter time delay from symptom onset to first medical contact [11,19]. However, approximately 30% of the young group presented late, more than six hours after symptom onset. This fact points out the importance of health education programs for patients and their families to achieve shorter hospital delays, not only in older persons but also in younger individuals, who are supposed to be at lower risk.

Anterior wall MI is more common in the younger population than in adults, according to previous studies [11,14]. In the VALIANT study, it was observed in 69.7% of patients aged between 18 and 45 years and in 56.4% of patients over 65 years [14]. Our findings support this opinion, but no significant difference between the two age groups was established (p=0.822).

More patients in group 2 presented with acute heart failure Killip class >II than those in group 1 with borderline significance, supporting the findings of previous studies [11,20]. Rosengren et al. reported an increasing proportion of patients presenting with heart failure (Killip class III-IV) with increasing age [20]. In particular, multivariate analysis showed that Killip class III or IV during hospitalization could be an independent predictor for hospital morbidity (OR: 31.15, 95% CI: 7.22-137.06, p<0.001), as well as for the combination of in-hospital morbidity and mortality (OR: 42.15, 95% CI: 8.13-218.57, p<0.001) in young patients [12]. In fact, all young patients in our study presenting with Killip class>II died during the index hospitalization, supporting the abovementioned statement. Surprisingly, we did not find a statistically significant difference between the LVEF during the hospital stay between both groups, which was in contrast with data supporting the hypothesis that LVEF is higher in young AMI patients than in older patients [11]. However, our results regarding the difference in LVEF between the two groups were in accordance with the meta-analysis presented by Lei and Bin [8], who also did not establish any significant differences between different age groups [8]. A quarter of our young population had reduced LVEF, and this was not negligible. Lv et al. [11] defined LVEF together with the higher Killip class as independent predictors for in-hospital mortality in young patients [11]. Mitral insufficiency ≥II grade was most common in older patients. Laboratory analysis revealed that young patients had higher peak CK activity and CK-MB activity than older patients, which was in agreement with a previous study by Chua et al. [12]. This may be an indicator of the extent of myocardial necrosis.

Our young patients also demonstrated some particularities in procedural and angiographic features, suggesting that young individuals need to be recognized as a specific AMI population with their individual profiles. The radial approach was the preferred approach in all patients but with a significantly higher prevalence in group 1. IRA was LAD in the majority of young cases, followed by RCA. For group 2, the RCA and LAD had almost the same incidence. The prevalence of LAD as a culprit artery in young AMI individuals has been previously reported in the literature [2,11,21]. In our study, in only one patient from the young age group, LMA was the target vessel.

Our data agree with previous reports that SVD is significantly more frequent among young MI patients than among their older counterparts (p<0.001) [2,8,16,20,21]. MVD and LMA involvement was a rare finding in group 1, and young patients tended to have less extended coronary atherosclerosis and less evolved CAD. Chen et al. [22] considered that the less severe coronary artery involvement and the fact that AMI in young patients is often not preceded by angina symptoms support the hypothesis that premature coronary artery disease is associated with rapid disease progression (plaque rupture and/or plaque complication) rather than a gradually developing process. They support this opinion with histopathological findings that revealed that atherosclerotic plaques in young patients with CAD are characterized by lipid-rich cores and a relative lack of fibrous caps [22]. On the other hand, myocardial infarction with nonobstructive coronary arteries (MINOCA) is found to be more common in young individuals than MI due to atherosclerotic CAD [23]. Therefore, pathophysiological mechanisms such as coronary vasospasm, coronary artery dissection, coronary embolism and AMI due to coronary anomalies are considered to be more frequent in younger patients [23]. In our young age group, 11.5% of patients had no evidence of significant coronary artery disease on angiography vs. only 1.8% of the older group (p=0.006). One of the young patients with NOCD had documented coronary spasm, one had Takotsubo cardiomyopathy, and in the rest five, the reason remained unclear. This highlights the necessity of additional investigations, such as intravascular ultrasound (IVUS) and optical coherence tomography (OCT), in patients with AMI and “normal” coronary angiography. According to current recommendations, invasive reperfusion therapy is the treatment of choice for STEMI and high-risk NSTEMI patients. We performed PCI procedures when appropriate after the assessment of the IRA. We registered high procedural success, over 90% in the two groups. The mean number of implanted stents was lower in the younger age group and thus supports the finding of less extensive coronary atherosclerosis in these patients.

It is widely accepted that in-hospital complications are more frequent among elderly patients, and the risk increases with age [20,24]. Consistent with previous studies, we found a significantly lower rate of in-hospital complications in our young patients (p=0.009) [4,12]. Notably, only postinfarction angina during the hospital stay was more frequent in the younger age group. When we focused only on the young AMI group, the established similarity correlated with data reported by Malik et al. [25], who found 29% in-hospital events in their study population.

The majority of authors define AMI in young persons as a benign condition with a relatively favorable prognosis. The in-hospital mortality rate in different series varies between 0.7% and 7% and is lower compared with older groups [12,15,20,26]. According to Canto et al. [27], the in-hospital mortality is even higher (10.2%) in young women under 45 years old with AMI, presenting without chest pain. Chua et al. [12] defined Killip classes III and IV as predictors of in-hospital morbidity and mortality in young patients. Gulati et al. [28] argue that the prognosis of young MI patients is not as benign, citing the similar incidence of adverse CV events in patients with and without significant coronary obstruction and the higher mortality of young MI patients compared with age- and gender-matched controls. Most likely, the in-hospital outcomes depend partially on the selected treatment strategy and the definition of “young” varying in different studies.

Our findings suggest that young patients have lower in-hospital mortality than their older counterparts (4.9% vs. 10.8%), but the difference was not significant (p=0.190). These results are consistent with those reported by Essilfie et al. [29]. However, this rate of in-hospital mortality seems to be higher than the most commonly mentioned rates of 2%-3% [11,25].

Several limitations exist and need to be mentioned: 1) This study represents a relatively small sample size, and a larger study needs to be performed in the future to confirm our findings. 2) There is a lack of data regarding the presence of hypercoagulable states or familial hypercholesterolemia in young AMI patients. 3) The COVID-19 pandemic may partially affect the morbidity and mortality rate in unrecognized individuals, as some patients are treated in this period. 4) IVUS or OCT was not used and can be a subject for future research.

## Conclusions

Bulgaria is a region with high CV morbidity and burden of mortality. However, there is a lack of data concerning the particularities of young patients with AMI in this country, and considering the long life expectancy of these patients, a careful analysis of this group is crucial. This retrospective study aimed to reveal the particularities of this uncommon entity.

We found a high prevalence of modifiable CV RF, such as smoking, dyslipidemia and AH, in the young group, less evolved CAD and comparably high procedural success between the two age groups. Approximately 30% of our young patients tended to present late in the hospital. The in-hospital mortality in the young population was lower than that in the older population but was still relatively higher than that previously reported. These findings highlight the necessity of early recognition of modifiable RF, improved primary and secondary prevention, initiation of education programs for young persons to shorten the hospital delay, improved access to health care, high awareness of clinicians in recognition of this condition and more effective treatment of this particular population group to reduce disability and premature mortality from CVD.
